# Structurally related Ca^2+^-release activated Ca^2+^ channel inhibitors exhibit distinct extracellular and intracellular modes of action

**DOI:** 10.1098/rsos.261544

**Published:** 2026-11-11

**Authors:** Yasin Gökçe, Yu Zhu, Sinayat Mahzabeen, Joshua Ghoorahoo, Matthew T. Harper, Nazmi Yaraş, Taufiq Rahman

**Affiliations:** ^1^ Department of Biophysics, Alanya Alaaddin Keykubat University, Alanya, Antalya, Turkey; ^2^ Department of Pharmacology, University of Cambridge, Cambridge, UK; ^3^ Department of Biophysics, Akdeniz University, Antalya, Turkey

**Keywords:** Ca^2+^-release activated Ca^2+^, Pyr6, BTP2, Synta66, GSK-7975A, molecular docking

## Abstract

Ca^2+^-release activated Ca^2+^ (CRAC) channels, formed by stromal interaction molecule (STIM) and Orai proteins, mediate store-operated Ca^2+^ entry and are implicated in autoimmune diseases, acute pancreatitis and cancer. Some small molecule inhibitors of these channels such as BTP2, Pyr6, Synta66 and GSK-7975A, are widely used as experimental tools, yet their precise mechanisms of action remain incompletely understood. Using whole-cell patch-clamp electrophysiology in rat basophilic leukaemia (RBL-1) cells, we determined whether each of these inhibitors acts from the extracellular or intracellular side of the plasma membrane. We found that BTP2 and GSK-7975A could suppress CRAC currents only when applied extracellularly, whereas Pyr6 and Synta66 were equally effective from either side. Molecular docking and molecular dynamics simulations using a rat Orai1 channel model identified plausible extracellular binding sites for all four inhibitors and additional putative intracellular binding sites for Pyr6 and Synta66. These predicted sites are located near regions that are likely to influence pore geometry and channel gating. Our findings suggest that, despite considerable structural similarity, these popular CRAC channel inhibitors exhibit mechanistically distinct interactions with Orai1, with important implications for their use as research tools and for future CRAC channel drug discovery.

## 1. Introduction

Ca^2+^-release activated Ca^2+^ (CRAC) channels are ubiquitous and highly Ca^2+^-selective ion channels that mediate the store-operated Ca^2+^ entry (SOCE) following physiological or pharmacological depletion of intracellular Ca^2+^ stores [1]. Their functional uniqueness arises from two distinctive features: an unusual activation mechanism requiring depletion of endoplasmic reticulum (ER) Ca^2+^ stores and their assembly on demand at ER–plasma membrane (ER–PM) junctions. A functional CRAC channel consists of the ER membrane-resident stromal interaction molecule (STIM) proteins, which sense the Ca^2+^ level within the ER lumen and, upon store depletion, interact with the PM-localized Orai proteins that form the Ca^2+^-conducting pore [2]. The resulting Ca^2+^ influx not only replenishes depleted intracellular stores but also governs specific cellular processes, including gene transcription, differentiation, proliferation, migration and exocytosis [1,2].

Although CRAC channels were initially considered to be a feature of non-excitable cells [3,4], it is now established that they are present in virtually all cell types, including neurons of both the central and peripheral nervous system. However, not all cell types rely on them to the same extent. Immune cells, particularly the T lymphocytes, manifest prominent SOCE, and properly functioning CRAC channels are indispensable for their proliferation and production of cytokines [1]. Excessive SOCE via hyperactive CRAC channels, often driven by overexpression of STIM and/or Orai proteins, underlies the pathophysiology of a range of autoimmune and inflammatory diseases [5,6]. Accordingly, CRAC channels have attracted considerable pharmaceutical interest, with companies including CalciMedica, Rhizen Pharmaceuticals, Vivreon Biosciences and ChemiCare focusing on developing CRAC inhibitors for conditions such as acute pancreatitis, rheumatoid arthritis, psoriasis, asthma and certain cancers. Additional areas, including stroke, acute renal injury, chronic pain, vascular remodelling and arterial thrombosis, are also being explored as potential indications for CRAC targeted therapies [6].

SOCE has been studied extensively since its first electrophysiological characterization in the early 1990s [3,7], and efforts to identify pharmacological modulators have proceeded in parallel [5,6]. Since the molecular identity of CRAC channel components—STIM and Orai proteins, was not established until 2005–2006 [8–12], early small molecule SOCE inhibitors were discovered primarily through phenotypic screens. The latter included functional readouts such as Ca^2+^ imaging, whole-cell electrophysiology or nuclear translocation of the transcription factor NFAT (nuclear factor of activated T cells). These campaigns yielded several chemically distinct SOCE-inhibitory scaffolds such as imidazoles (e.g. SKF-96365), boronates (e.g. 2-APB and derivatives) and bis(trifluoromethyl)pyrazoles (BTPs). Among these compounds, a pyrazole compound—BTP2 (also known as YM-58483) is one of the most widely used inhibitors of SOCE and CRAC channel function. Related or subsequently developed inhibitors such as Synta66, GSK-7975A and Pyr6 are also frequently used as experimental tools to suppress SOCE or CRAC channel activity [5]. Despite their widespread use, the precise mechanism(s) by which these molecules inhibit CRAC channels remain incompletely understood. For some of these compounds, previous studies have suggested direct or allosteric effects on the Orai pore rather than disruption of STIM–Orai coupling [13,14]. In particular, Waldherr *et al.* [14] identified an extracellular Synta66-binding region involving TM1/TM3 and extracellular loops 1 and 3, close to the Orai1 selectivity filter. More recently, GSK-7975A-derived photo-crosslinking probes provided evidence for direct interaction with hOrai1, although the precise binding site could not be established experimentally [15]. Thus, the precise binding sites and modes of interaction of these widely used CRAC inhibitors remain incompletely resolved. This uncertainty is important because these inhibitors are often used interchangeably as tool compounds [16], even though differences in their mechanisms could substantially affect interpretation of experimental results.

In this study, we used whole-cell patch-clamp electrophysiology in rat basophilic leukaemia-1 (RBL-1) cells measuring *I*_CRAC_ (the characteristic whole-cell current representing SOCE/active CRAC channels) [1] to determine whether four widely used CRAC channel inhibitors—BTP2, Pyr6, Synta66 and GSK-7975A act from the extracellular or intracellular face of the PM. RBL-1 cells were chosen because they represent a well-established mast-cell model that exhibits robust endogenous CRAC currents and has been extensively used for electrophysiological studies of SOCE [1,4]. Moreover, mast cells were the cellular system in which *I*_CRAC_ was originally characterized [7].

For comparison of the four CRAC channel inhibitors, we used a fixed concentration of 10 µM. Although this concentration is relatively high compared with the reported potencies of some of these compounds, 10 µM has previously been used to achieve substantial or maximal CRAC/SOCE inhibition [13,15–17]. Reported inhibitor potencies can also vary depending on the cellular and experimental context [16–18]. We deliberately selected this concentration because our primary objective was not to compare inhibitor potency, but to determine whether inhibition could be achieved following extracellular versus intracellular application. Use of a common, strongly inhibitory concentration therefore facilitated comparison of the sidedness of action among the four compounds.

Despite the comparable physico-chemical properties across the four chosen CRAC inhibitors (table 1), we here identify significant differences in their possible sites of action. We then complement these functional findings with *in silico* modelling, molecular docking and molecular dynamics (MD) simulations of the rat Orai1 hexamer to propose putative binding sites and modes of interaction for each inhibitor.

**Table 1.** Molecular mechanics/Poisson–Boltzmann surface area (MM/PBSA) binding free energies (kcal mol^–1^) of the selected CRAC channels inhibitors.

system	ΔVDWAALS	ΔEEL	ΔEPB	ΔENPOLAR	ΔGGAS	ΔGSOLV	ΔTOTAL	ΔG binding
BTP2	−34.98 ± 2.58	−5.30 ± 3.93	22.19 ± 5.33	−3.97 ± 0.16	−40.28 ± 4.17	18.22 ± 5.33	−22.05 ± 5.16	−13.87 ± 5.16
GSK-7975A	−35.53 ± 2.21	−19.37 ± 4.01	35.71 ± 5.00	−4.14 ± 0.10	−54.91 ± 4.71	31.58 ± 4.98	−23.32 ± 5.42	−15.54 ± 5.42
Pyr6_out_	−34.67 ± 2.63	−10.19 ± 4.74	16.34 ± 4.50	−3.94 ± 0.17	−44.86 ± 4.56	12.40 ± 4.43	−32.46 ± 4.02	−24.21 ± 4.02
Pyr6_in_	−37.99 ± 2.29	−0.63 ± 3.68	20.13 ± 5.07	−4.33 ± 0.10	−38.62 ± 4.38	15.79 ± 5.04	−22.83 ± 5.97	−15.26 ± 5.97
Synta66_out_	−38.31 ± 3.38	−14.22 ± 6.41	26.99 ± 5.12	−4.26 ± 0.16	−52.53 ± 7.26	22.74 ± 5.05	−29.79 ± 5.24	−17.31 ± 6.42
Synta66_in_	−33.59 ± 2.38	−9.96 ± 4.11	20.35 ± 3.50	−4.20 ± 0.12	−43.55 ± 4.69	16.15 ± 3.44	−27.40 ± 4.60	−14.83 ± 4.72

*Notes:* All the values in the table are represented in mean ± s.d. The subscripts ‘out’ and ‘in’ refer to the extracellular and intracellular binding modes, respectively, for Pyr6 and Synta66.

Abbreviations: ΔVDWAALS: van der Waals contribution; ΔEEL: electrostatic contribution; ΔEPB: polar contribution to the solvation free energy for PB calculations; ΔENPOLAR: non-polar contribution to the solvation free energy for PB calculations; ΔGGAS: interaction energy, sum of the bonded and the non-bonded energy; ΔGSOLV: the sum of the polar and non-polar contributions; ΔTOTAL: changes in total energy; ΔG binding: estimated binding free energy for protein–ligand complex (kcal mol^−1^).

## 2. Results and discussion

### 2.1. Evaluation of the effects of chosen inhibitors on *I*_CRAC_

We first examined BTP2 (figure 1A)—a prototypical member of the BTP class and one of the most widely used inhibitors of SOCE/CRAC channels [5]. This molecule was first identified in 2000 by scientists from Abbot Laboratories as inhibitors of interleukin-2 (IL-2) synthesis in a high throughput reporter (luciferase) gene-based screen of Jurkat T cells with an overall goal to find better, non-macrocyclic immunosuppressants [19]. Members of the BTP series were found to manifest good structure–activity relationship in retarding nuclear translocation of NFAT and thus reducing IL-2 production, although little was then known about the underlying molecular mechanism [19]. BTP2 was later shown to inhibit SOCE [20] and CRAC channels [21].

**Figure 1.**
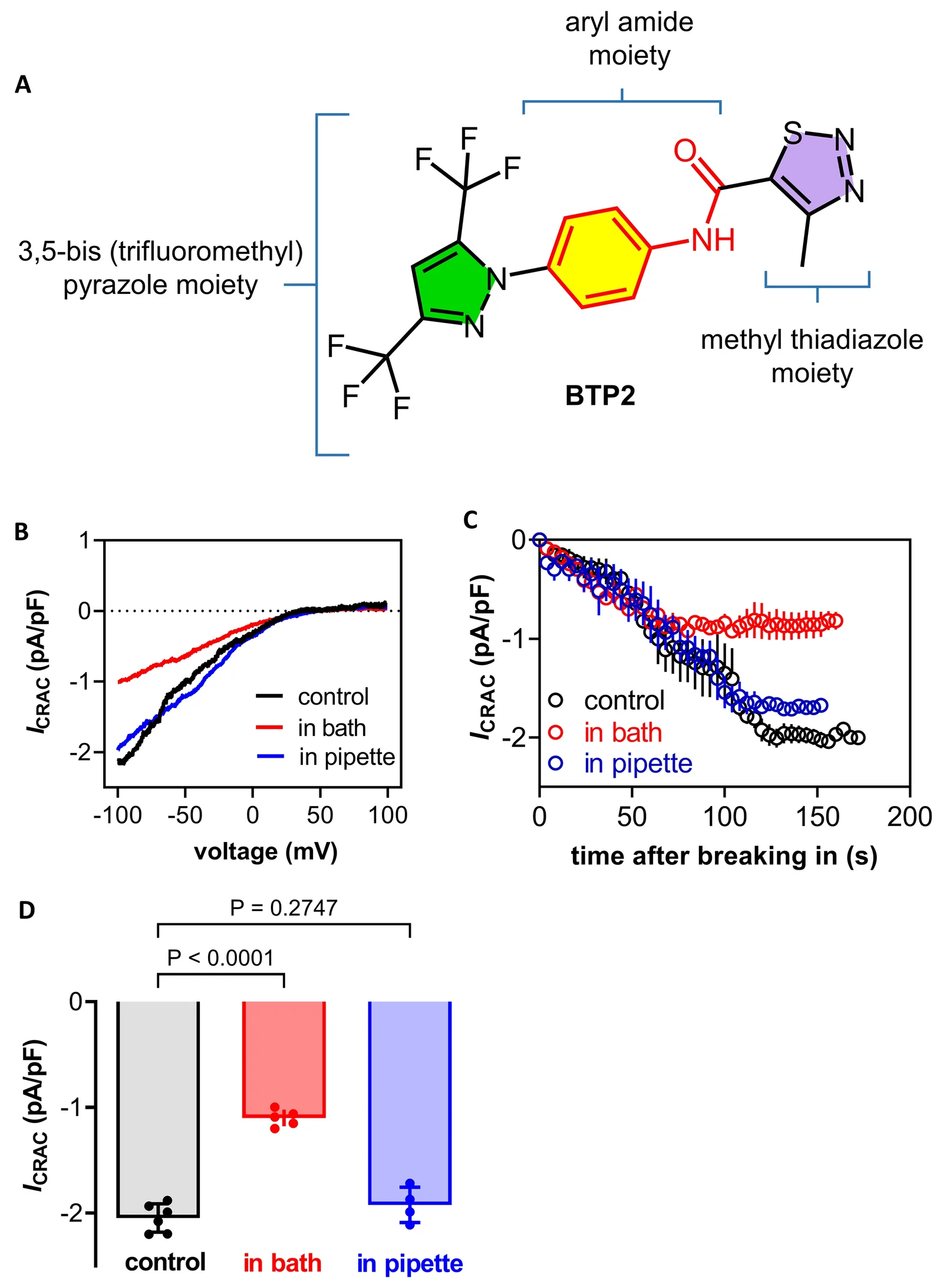
Effects of BTP2 on CRAC channel function in RBL-1 cells. Prior to recording of the whole-cell current representing CRAC channel function (*I*_CRAC)_, RBL-1 cells were pretreated with BTP2 (10 µM) for approximately 20 minutes added to the bath solution or included in the pipette solution while control cells were treated with dimethyl sulfoxide (DMSO) only. (A) Two-dimensional chemical structure of BTP2, highlighting different substructural features. (B) Typical current–voltage (I–V) relationships of *I*_CRAC_ (pA) evoked by voltage ramps from −100 to +100 mV, normalized to cell size (pF) under different experimental conditions. (C) Representative peak *I*_CRAC_ size (as current density, pA pF^−1^) versus time plots (each data point as mean ± s.e.m.) from whole-cell recording of at least four different cells (*n* = 4) in each condition are shown. (D) Bar graphs (mean ± s.d.) showing current densities at −80 mV holding potential under each condition. The statistical comparisons with respect to the control were done using one-way ANOVA followed by Dunnett's test; *P*-values are shown above bars.

As can be seen in figure 1B–D, BTP2 when added to the extracellular (bath) solution at 10 µM, significantly suppressed the peak *I*_CRAC_ current. By contrast, inclusion of BTP2 at the same concentration in the intracellular (pipette) solution produced only a modest, statistically non-significant reduction in peak *I*_CRAC_ size. These data support a predominantly extracellular mode of action for BTP2. This is consistent with previous findings in Jurkat T cells, where acute BTP2 application after Ca^2+^ entry had plateaued was as effective as prolonged pre-incubation, leading the authors to propose a direct extracellular interaction with channel mediating the SOCE [20].

Although a contemporaneous study [21] using similar cells differed significantly in finding the potency and the kinetics of SOCE blockade upon acute challenge of BTP2, it did confirm extracellular site of action of this CRAC channel inhibitor since its inclusion within the intracellular solution (at 1 µM) did not affect *I*_CRAC_. Our finding with RBL-1 cells about BTP2′s extracellular site of action therefore strongly aligns with these observations in Jurkat T and related immune cells [20,21].

We next examined Pyr6 (figure 2A), a BTP-class SOCE inhibitor that is structurally closely related to BTP2, differing only in the replacement of the methylthiadiazole moiety with a fluoropyridine group [5]. This single modification, probably representing a bioisosteric replacement, has been shown to confer markedly greater selectivity and potency for CRAC channels over TRPC3 channels [22]. Despite this close structural resemblance to BTP2, Pyr6 at 10 µM behaved in striking contrast in our whole-cell recordings of RBL-1 cells: it significantly inhibited *I*_CRAC_ when applied from either side of the PM (figure 2B–D). This suggests that the fluoropyridine moiety, despite being a relatively minor structural change, fundamentally alters the site or mode of interaction with the CRAC channel.

**Figure 2.**
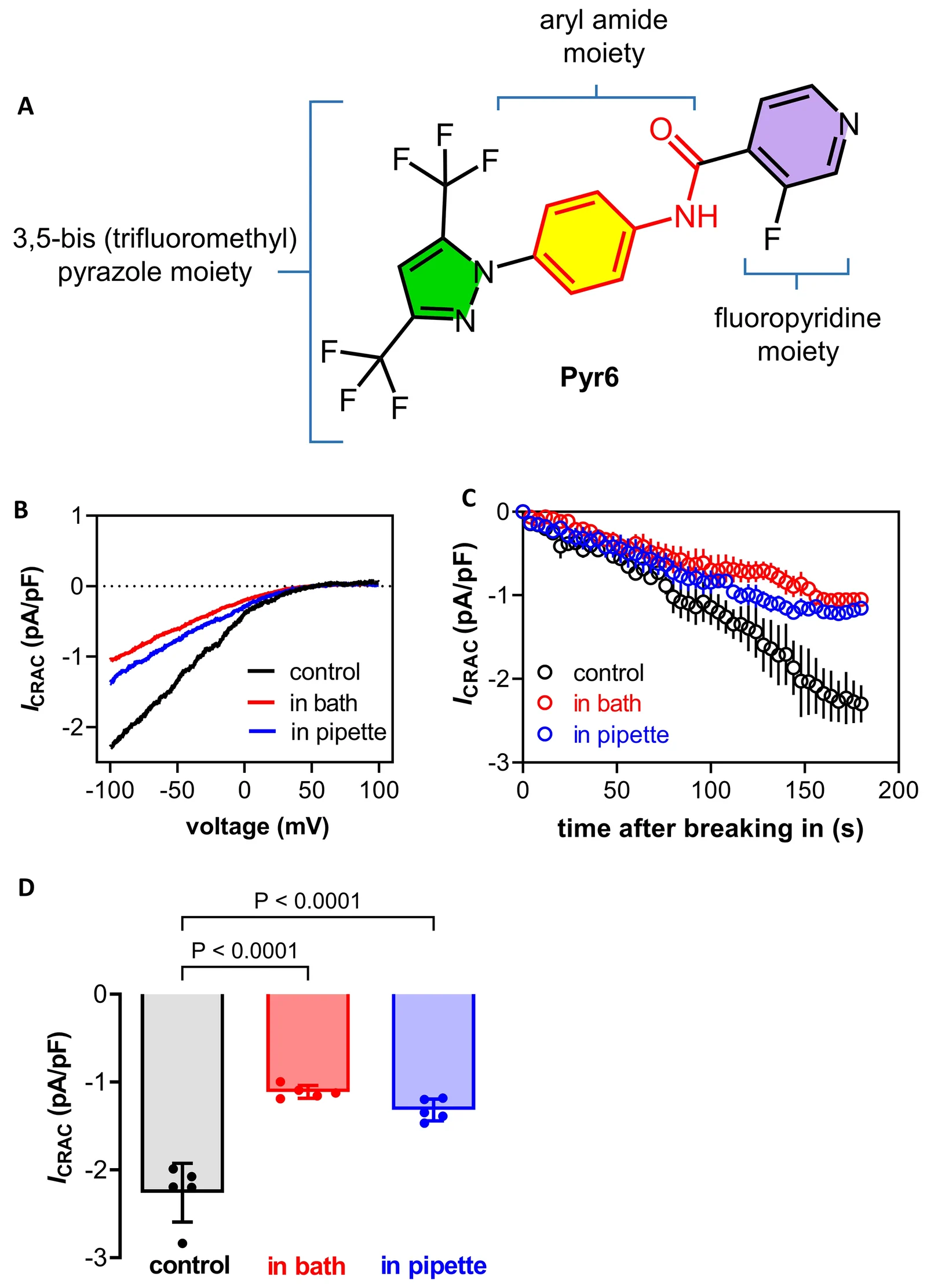
Effects of Pyr6 on CRAC channel function in RBL-1 cells. Prior to recording of the whole-cell current representing CRAC channel function (*I*_CRAC_), RBL-1 cells were pretreated with Pyr6 (10 µM) for approximately 20 minutes added to the bath solution or included in the pipette solution while control cells were treated with DMSO only. (A) Two-dimensional chemical structure of Pyr6, highlighting different substructural features. (B) Typical current–voltage (I–V) relationships of *I*_CRAC_ (pA) evoked by voltage ramps from −100 to +100 mV, normalized to cell size (pF) under different experimental conditions. (C) Representative peak *I*_CRAC_ size (as current density, pA pF^−1^) versus time plots (each data point as mean ± s.e.m.) from whole-cell recording of at least four different cells (*n* = 4) in each condition are shown. (D) Bar graphs (mean ± s.d.) showing current densities at −80 mV holding potential under each condition. The statistical comparisons with respect to the control were done using one-way ANOVA followed by Dunnett's test; *P*-values are shown above bars.

GSK-7975A (figure 3A) is a member of the N-pyrazole carboxamide chemical series developed by GlaxoSmithKline (GSK patent WO/2010/122089) and is also well-recognized as an effective inhibitor of CRAC channels [5]. Mechanistically, it has been shown to act as an allosteric blocker of the Orai pore, without disrupting STIM –STIM or STIM–Orai interactions and with an affinity that is sensitive to the geometry of the selectivity filter [13]. From two-dimensional chemical structural point of view, GSK-7975A appears to be distinct from the BTP2 and Pyr6 (figure 3A). Despite this structural divergence, when tested at 10 µM in whole-cell recordings of RBL-1 cells, GSK-7975A closely replicated the behaviour of BTP2, significantly suppressing *I*_CRAC_ only when applied from the extracellular side of the PM (figure 3). This functional convergence between two structurally dissimilar compounds is noteworthy and suggests that, despite differences in their chemical scaffolds, both BTP2 and GSK-7975A share a common extracellular site of action on the CRAC channel.

**Figure 3.**
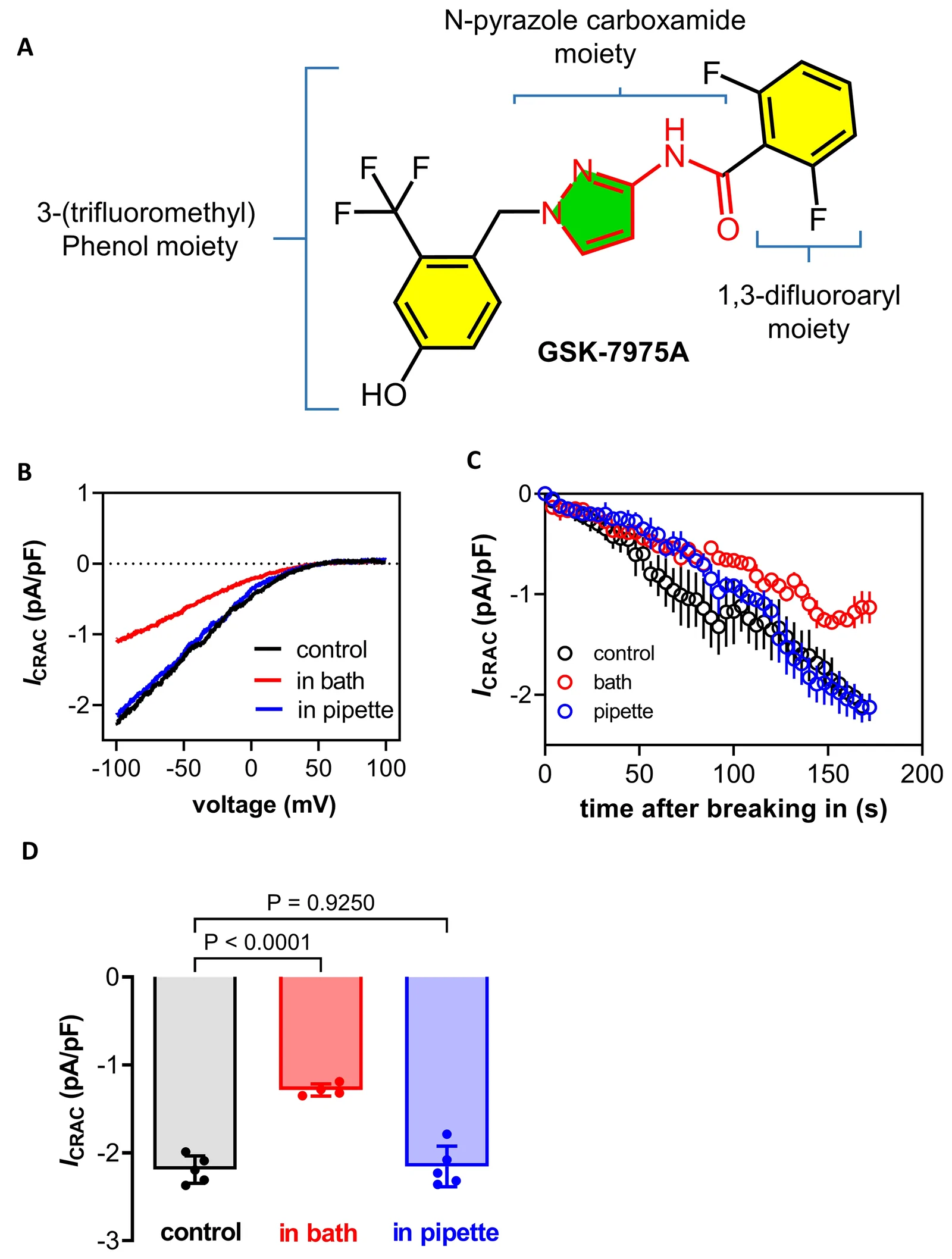
Effects of GSK-7975A on CRAC channel function in RBL-1 cells. Prior to recording of the whole-cell current representing CRAC channel function (*I*_CRAC_), RBL-1 cells were pretreated with GSK-7975A (10 µM) for approximately 20 minutes added to the bath solution or included in the pipette solution while control cells were treated with DMSO only. (A) Two-dimensional chemical structure of GSK-7975A, highlighting different substructural features. (B) Typical current–voltage (I–V) relationships of *I*_CRAC_ (pA) evoked by voltage ramps from −100 to +100 mV, normalized to cell size (pF) under different experimental conditions. (C) Representative peak *I*_CRAC_ size (as current density, pA pF^−1^) versus time plots (each data point as mean ± s.e.m.) from whole-cell recording of at least four different cells (*n* = 4) in each condition are shown. (D) Bar graphs (mean ± s.d.) showing current densities at −80 mV holding potential under each condition. The statistical comparisons with respect to the control were done using one-way ANOVA followed by Dunnett's test; *P*-values are shown above bars.

Synta66 is a selective inhibitor of SOCE and CRAC channel activity, developed by Synta Pharmaceuticals, now merged into Madrigal Pharmaceuticals [23]. It does not appear to affect STIM protein clustering in vascular smooth muscle cells [24], and recent work suggests that it acts as an allosteric blocker of the Orai1 pore, similar to GSK-7975A [14]. Structurally, Synta66 more closely resembles Pyr6, differing mainly by replacement of the 3,5-bis(trifluoromethyl)pyrazole ring with a 2,5-dimethoxybenzene ring (figure 4A).

**Figure 4.**
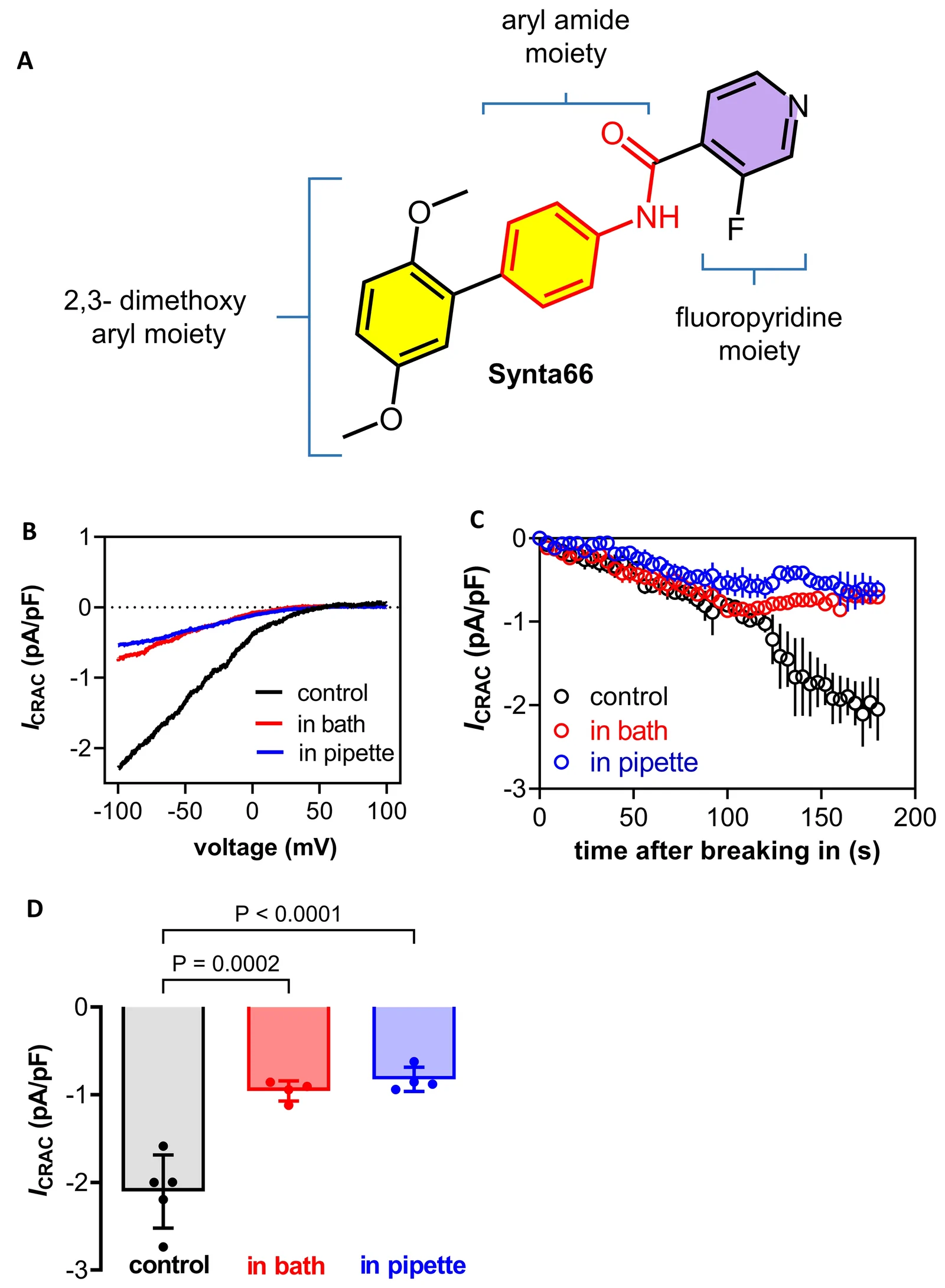
Effects of Synta66 on CRAC channel function in RBL-1 cells. Prior to recording of the whole-cell current representing CRAC channel function (*I*_CRAC_), RBL-1 cells were pretreated with Synta66 (10 µM) for approximately 20 minutes added to the bath solution or included in the pipette solution while control cells were treated with DMSO only. (A) Two-dimensional chemical structure of Synta66, highlighting different substructural features. (B) Typical current-voltage (I–V) relationships of *I*_CRAC_ (pA) evoked by voltage ramps from −100 to +100 mV, normalized to cell size (pF) under different experimental conditions. (C) Representative peak *I*_CRAC_ size (as current density, pA pF^−1^) versus time plots (each data point as mean ± s.e.m.) from whole-cell recording of at least four different cells (*n* = 4) in each condition are shown. (D) Bar graphs (mean ± s.d.) showing current densities at −80 mV holding potential under each condition. The statistical comparisons with respect to the control were done using one-way ANOVA followed by Dunnett's test; *P*-values are shown above bars.

Consistent with this structural similarity to Pyr6, and in contrast to GSK-7975A with which it shares a proposed allosteric mechanism, Synta66 at 10 µM showed a bidirectional inhibitory profile of Pyr6, significantly suppressing *I*_CRAC_ in RBL-1 cells when applied from either the extracellular or intracellular side of the PM (figure 4B–D), with comparable efficacy in both conditions. This finding does not necessarily exclude an allosteric mechanism of Orai inhibition, but suggests that Synta66 may access functionally relevant interaction site(s) from either side of the membrane.

Taken together, our electrophysiological evaluation of all four inhibitors reveals a clear mechanistic divide: BTP2 and GSK-7975A suppress *I*_CRAC_ exclusively from the extracellular side, whereas Pyr6 and Synta66 are equally effective from either side of the membrane. Notably, this functional divergence cannot be readily attributed to differences in membrane permeability, given that all four compounds share broadly comparable physico-chemical properties (supplementary material, table S1), including similar molecular weights, lipophilicity (XlogP3) and topological polar surface area (TPSA). This unexpected mechanistic divergence among structurally related compounds prompted us to investigate their putative binding sites and modes of interaction with the Orai1 protein using *in silico* molecular docking and MD simulations.

### 2.2. Putative binding modes of selected Ca^2+^-release activated Ca^2+^ inhibitors on rat Orai1 hexamer

For unbiased prediction of the putative binding site(s) and binding mode(s) of the selected CRAC inhibitors evaluated electrophysiologically in this study, we used an *in silico* protocol that we described previously [25,26]. This involved initial blind docking using AutoDockVina [27] followed by focused docking using GOLD suite [28]. As the primary cell line used in our electrophysiological experiments (RBL-1 cells) is of rat origin, we first generated a homology model of the rat Orai1 (rOrai1) hexamer based on the published crystal structure of *Drosophila* Orai (PDB ID: 4HKR). A hexameric rOrai1 model was used in line with the broad consensus in the field that functional CRAC channels, in a physiological setting, comprise hexameric Orai proteins in complex with STIM proteins [1]. Following refinement through the GalaxyRefine2 server [29], the final rOrai1 model displayed acceptable quality attributes [30] including all-atoms MolProbity [30] score of 0.79, clash score of 0.00, 96.26% of the residues in Ramachandran favoured regions, 0.98% residues as Ramachandran outliers and 0.72% being poor rotamers (supplementary material, figure S2).

As the protein (rOrai1 hexamer) was held rigid during docking with both AutoDock Vina and GOLD, the best-scoring docked pose obtained for each CRAC inhibitor was subsequently subjected to all-atom MD simulations in explicit water, with the modelled rOrai1 hexamer embedded in a 1-palmitoyl,2-oleoyl-sn-glycero-3-phosphocholine (POPC) bilayer. This approach allowed further refinement and stability assessment of the docked poses, together with estimation of the free energy of binding using the molecular mechanics/Poisson–Boltzmann surface area (MM/PBSA) method.

#### 2.2.1. Putative binding mode of BTP2 on the rOrai1 hexamer

The most frequently sampled putative binding site for BTP2 in our *in silico* docking protocol was located between the pore-forming M1 helices of two adjacent rOrai1 subunits. In this pose, BTP2 partially occluded the Ca^2+^-permeation pathway. When a representative docked complex was subjected to MD simulation, BTP2 stabilized rapidly following a brief equilibration period. During the simulation, the ligand shifted to a different but equivalent pair of neighbouring M1 helices and adopted a stable orientation approximately parallel to the pore axis, as supported by the plateauing of the root-mean-square deviation (RMSD) profile (figure 5A,B).

**Figure 5.**
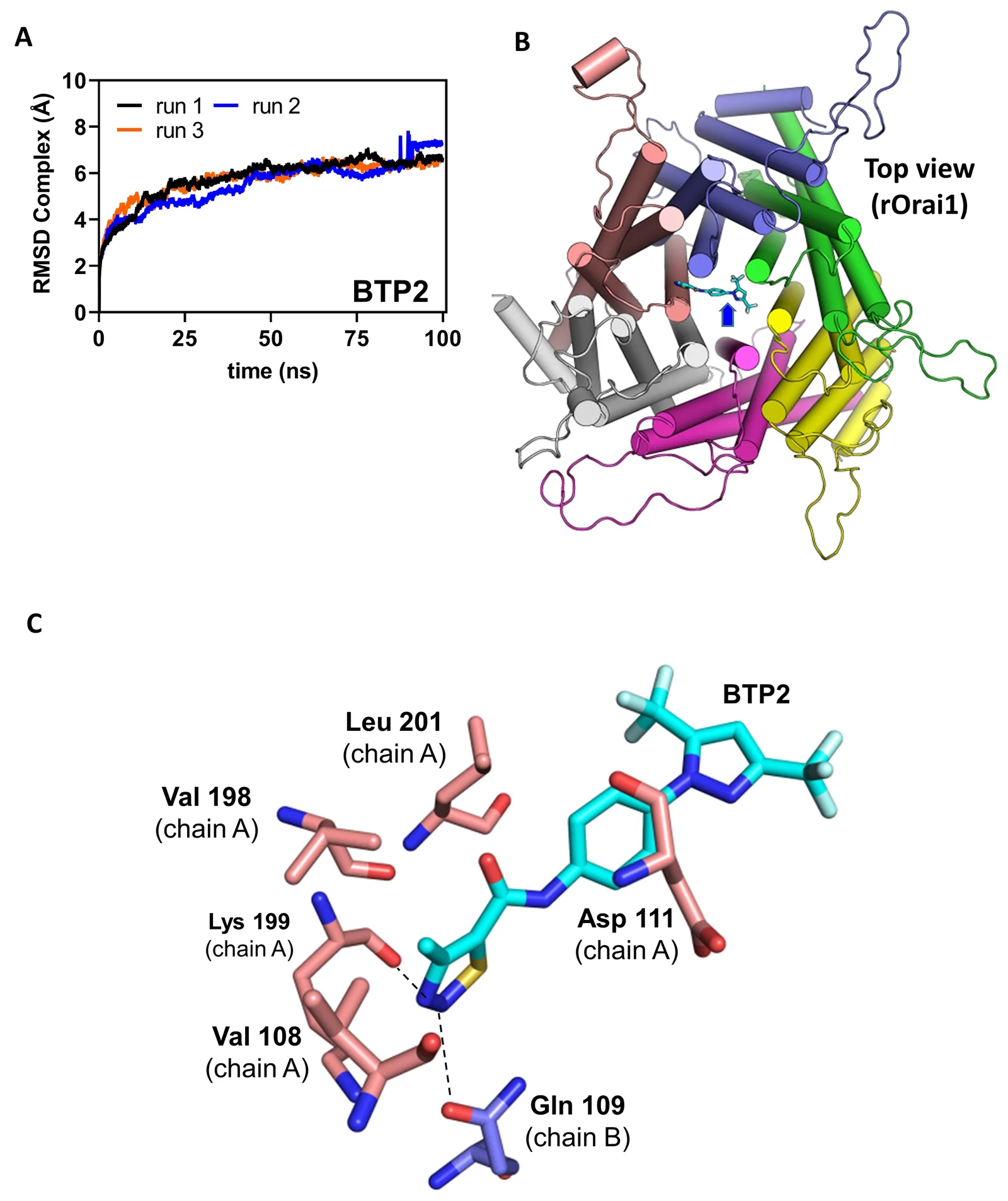
*In silico* prediction of probable binding site and binding mode of BTP2 on rOrai1 hexamer. (A) RMSD plot of BTP2-rOrai1 docked complex obtained from trajectory analyses of 100 ns of MD simulations performed in three independent runs. (B) Top view from the extracellular side showing the most stable pose of BTP2 (shown as stick representation) on rOrai1 model (shown as cylinders). The pose was obtained from MD simulations using predicted binding mode of BTP2 through initial blind docking with AutoDock Vina followed by focused docking using GOLD suite. The ligand pose is shown with a blue arrow. (C) Three-dimensional-ligand interaction profile of the typical best pose of BTP2 generated through PLIP server. The ligand and the proximal residues are shown as sticks. Hydrogen bonding interactions are shown with dashed lines. Figures were prepared using PyMOL.

The methylthiadiazole moiety of BTP2 progressively approached the protein surface, ultimately residing stably within a pocket at the subunit interface delineated by two neighbouring M1 helices and one M3 helix. Hydrogen bonds were observed between BTP2 and Gln109 and Lys199, each contributed by a different neighbouring subunit. The central aryl moiety engaged in hydrophobic interactions primarily with Asp111 and Leu201 of a single subunit, while an additional hydrophobic contact was noted between the methyl group of the methylthiadiazole moiety and Val198 (figure 5C). Collectively, these interactions appear to stabilize the predicted binding mode of BTP2 within the extracellular pore region of the rOrai1 model. The estimated energetic profile of the BTP2-rOrai1 interaction is shown in table 1.

#### 2.2.2. Putative binding mode of Pyr6 on the rOrai1 hexamer

Chemically, Pyr6 can be regarded as a structural hybrid of BTP2 and Synta66, in which the 2,3-dimethoxyaryl moiety of Synta66 is replaced by the 3,5-bis(trifluoromethyl)pyrazole moiety present in BTP2 (figure 2A). Functionally, Pyr6 resembled Synta66 but differed from BTP2, as it significantly suppressed *I*_CRAC​_ when applied either extracellularly or intracellularly. We therefore explored both extracellular and intracellular plausible binding sites for Pyr6 on the rOrai1 hexamer using our *in silico* docking workflow.

At the extracellular face of the rOrai1 hexamer, the most frequently obtained, high-ranking docked pose of Pyr6 was similar to that predicted for BTP2. In this pose, Pyr6 was positioned between the pore-forming M1 helices of two adjacent rOrai1 subunits, thereby partially spanning the extracellular pore region. Upon MD simulation of the corresponding complex, Pyr6 exhibited minimal RMSD fluctuations (figure 6A), consistent with stable occupancy within an interhelical pocket formed by two neighbouring M1 helices and an adjacent M3 helix from another subunit (figure 6B).

**Figure 6.**
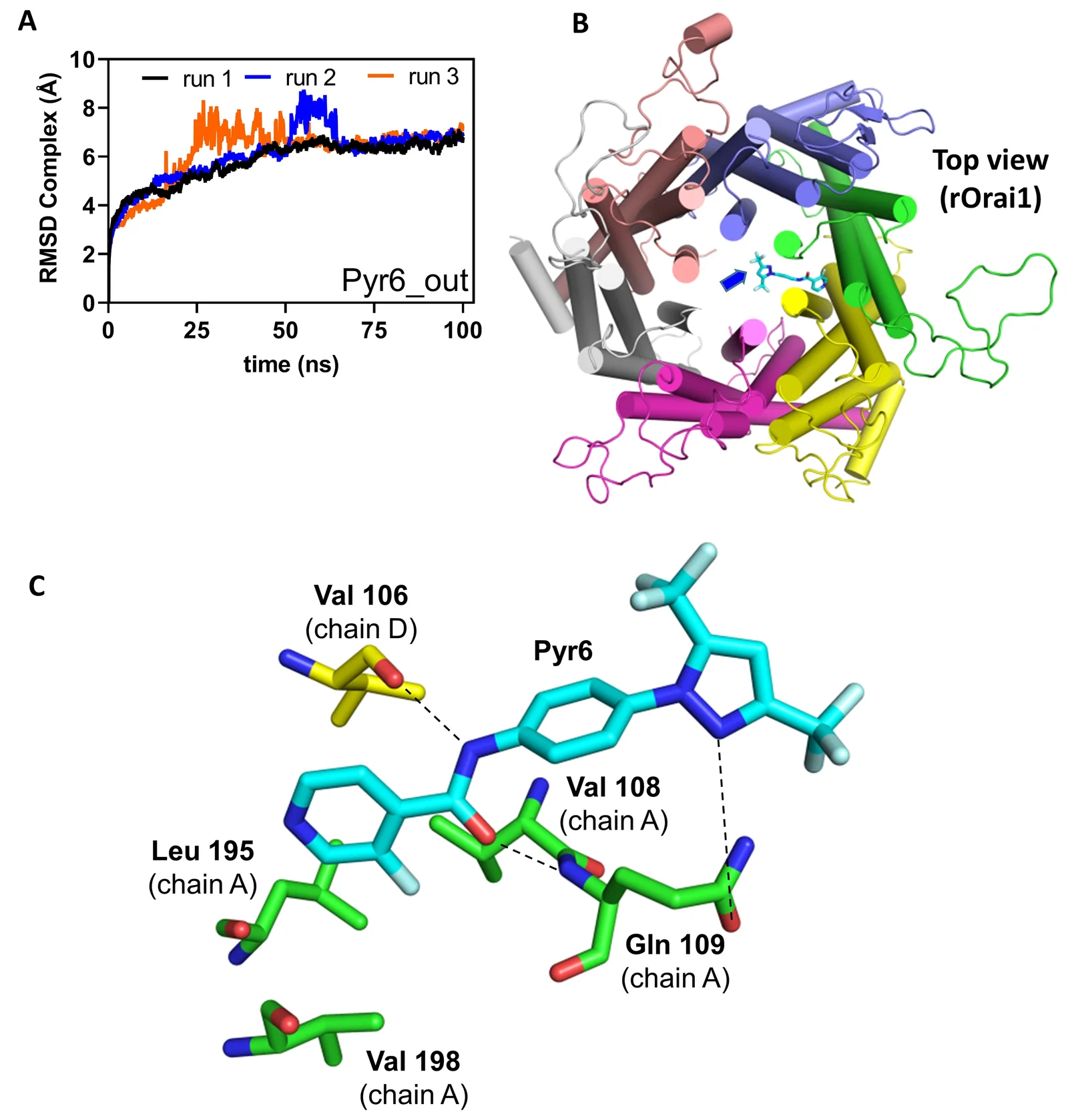
*In silico* prediction of probable binding site and binding mode of Pyr6 on the extracellular face of rOrai1 hexamer. (A) RMSD plot of Pyr6-rOrai1 docked complex obtained from trajectory analyses of 100 ns of MD simulations performed in three independent runs. (B) Top view from the extracellular side showing the most stable pose of Pyr6 (designated as Pyr6_out_, shown as stick representation) on the extracellular face of the rOrai1 model (shown as cylinders). The pose was obtained from MD simulations using predicted binding mode of Pyr6 through initial blind docking with AutoDock Vina followed by focused docking using GOLD suite. The ligand pose is shown with a blue arrow. (C) Three-dimensional-ligand interaction profile of the typical best pose of Pyr6_out_ generated through PLIP server. The ligand and the proximal residues are shown as sticks. Hydrogen bonding interactions are shown with dashed lines. Figures were prepared using PyMOL.

Protein–ligand interaction profiler (PLIP)-based analysis (figure 6C) revealed three hydrogen-bonding interactions: between the side-chain of Gln109 (one subunit) and a nitrogen atom of the fluoropyridine moiety of Pyr6; between the backbone amide nitrogen of the same Gln109 residue and the carbonyl oxygen of the aryl amide moiety of Pyr6; and between the backbone carbonyl oxygen of Val106 (a neighbouring subunit) and the amide nitrogen of the aryl amide moiety of Pyr6. In addition, the fluoropyridine moiety engaged in hydrophobic interactions with Leu195 and Val198 of one subunit, while Val108 of the same subunit made hydrophobic contact with the central aryl core of Pyr6. This combination of hydrogen bonds and hydrophobic contacts secured Pyr6 within the pocket, despite observable rotational adjustments among its aromatic rings. Notably, the BTP moiety gradually shifted closer to the pore axis over the course of the simulation, reflecting ligand dynamics oriented towards an energetically optimal binding configuration.

Because Pyr6 inhibited CRAC channel activity when also introduced intracellularly, we additionally explored a putative cytosolic binding site using blind docking with AutoDock Vina followed by focused docking with the GOLD suite. The resulting intracellular pose, designated Pyr6_in_, was further refined and assessed for stability by 100 ns MD simulations performed in three independent replicates.

Pyr6_in_ remained largely stable throughout the simulations and was localized within a distinct, narrow pocket near the cytosolic ends of the M1 and M3 helices (figure 7A,B). This deep, constricted pocket appeared to anchor Pyr6 through hydrogen bonding with Lys86 from one rOrai1 subunit, together with hydrophobic interactions involving Arg168, Arg171, Glu174 and Leu175 from the same subunit, as well as Leu146, Pro97 and Glu150 from a neighbouring subunit (figure 7C). Thus, the cytosolic pocket appears to create a structural bottleneck that limits the conformational flexibility of Pyr6 and stabilizes its intracellular binding pose.

**Figure 7.**
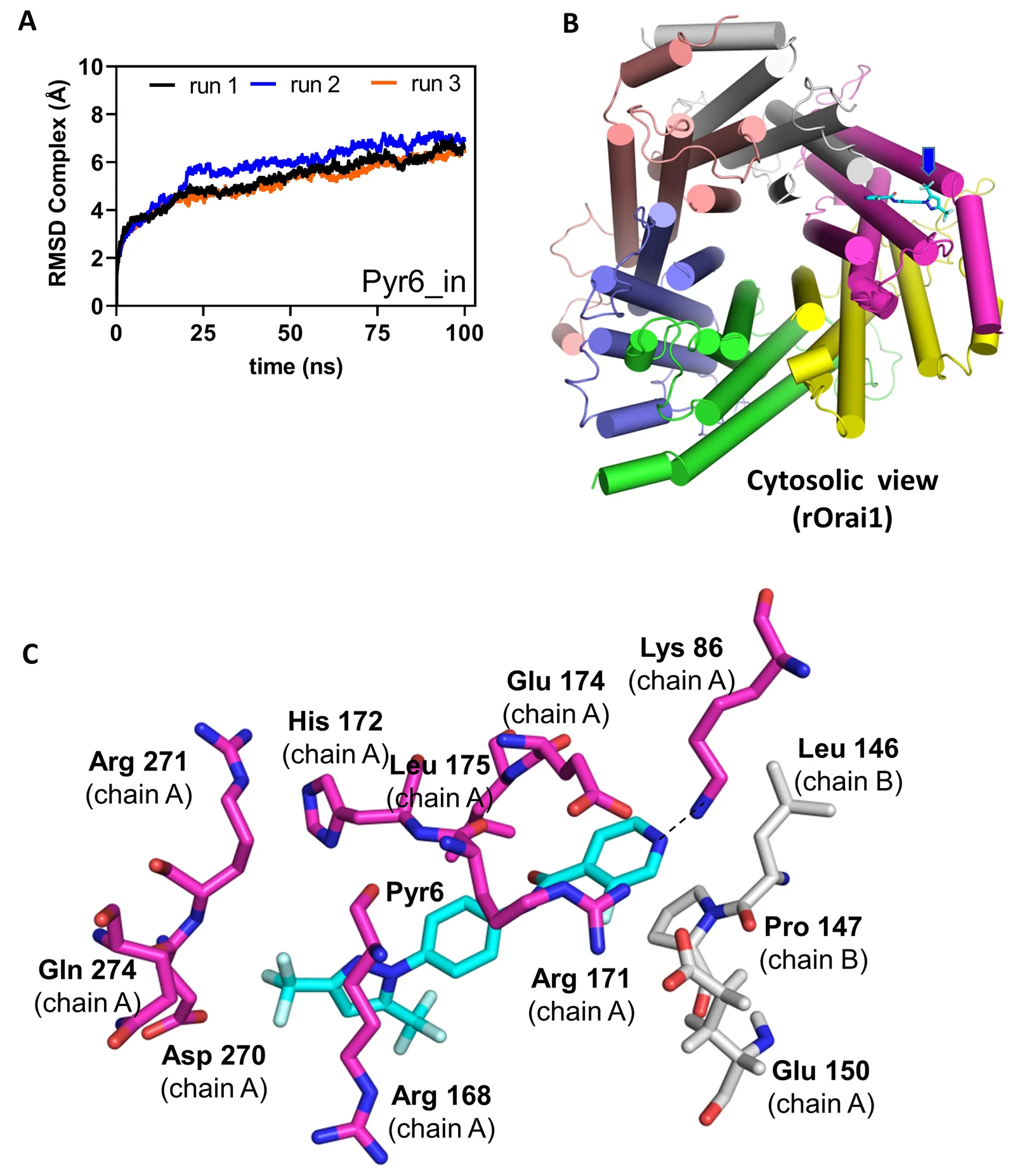
*In silico* prediction of probable binding site and binding mode of Pyr6 on the intracellular face of rOrai1 hexamer. (A) RMSD plot of Pyr6-rOrai1 docked complex obtained from trajectory analyses of 100 ns of MD simulations performed in three independent runs. (B) View from the intracellular side showing the most stable pose of Pyr6 (designated as Pyr6_in_, shown as stick representation) on the cytosolic face of the rOrai1 model (shown as cylinders). The pose was obtained from MD simulations using predicted binding mode of Pyr6 through initial blind docking with AutoDock Vina followed by focused docking using GOLD suite. The ligand pose is shown with a blue arrow. (C) Three-dimensional-ligand interaction profile of the typical best pose of Pyr6_in_ generated through PLIP server. The ligand and the proximal residues are shown as sticks. Hydrogen bonding interactions are shown with dashed lines. Figures were prepared using PyMOL.

The estimated energetic profiles of Pyr6 at the predicted extracellular and intracellular binding pockets are shown in table 1.

#### 2.2.3. Putative binding mode of GSK-7975A on the rOrai1 hexamer

GSK-7975A (figure 3A), which contains an intermediary pyrazole amide moiety, is chemically somewhat distinct from BTP2, Pyr6 and Synta66, all of which possess an aryl amide moiety flanking both ends of their structures. Like BTP2, this molecule inhibited CRAC channel activity in our whole-cell recordings only when applied extracellularly (i.e. in the bath solution; figure 3B). We therefore restricted our docking analysis for this inhibitor to possible extracellular binding site(s) only. The best-ranked extracellular pose was subsequently subjected to 100 ns MD simulations performed in triplicate. Analysis of the simulation trajectories showed that GSK-7975A maintained positional consistency throughout, as reflected by low RMSD fluctuations (figure 8A). Notably, the trifluoromethylphenol and pyrazole moieties remained in proximity to the M1 helices of adjacent rOrai1 subunits, while the difluoroaryl moiety was positioned adjacent to the M3 helix of one subunit (figure 8B).

**Figure 8.**
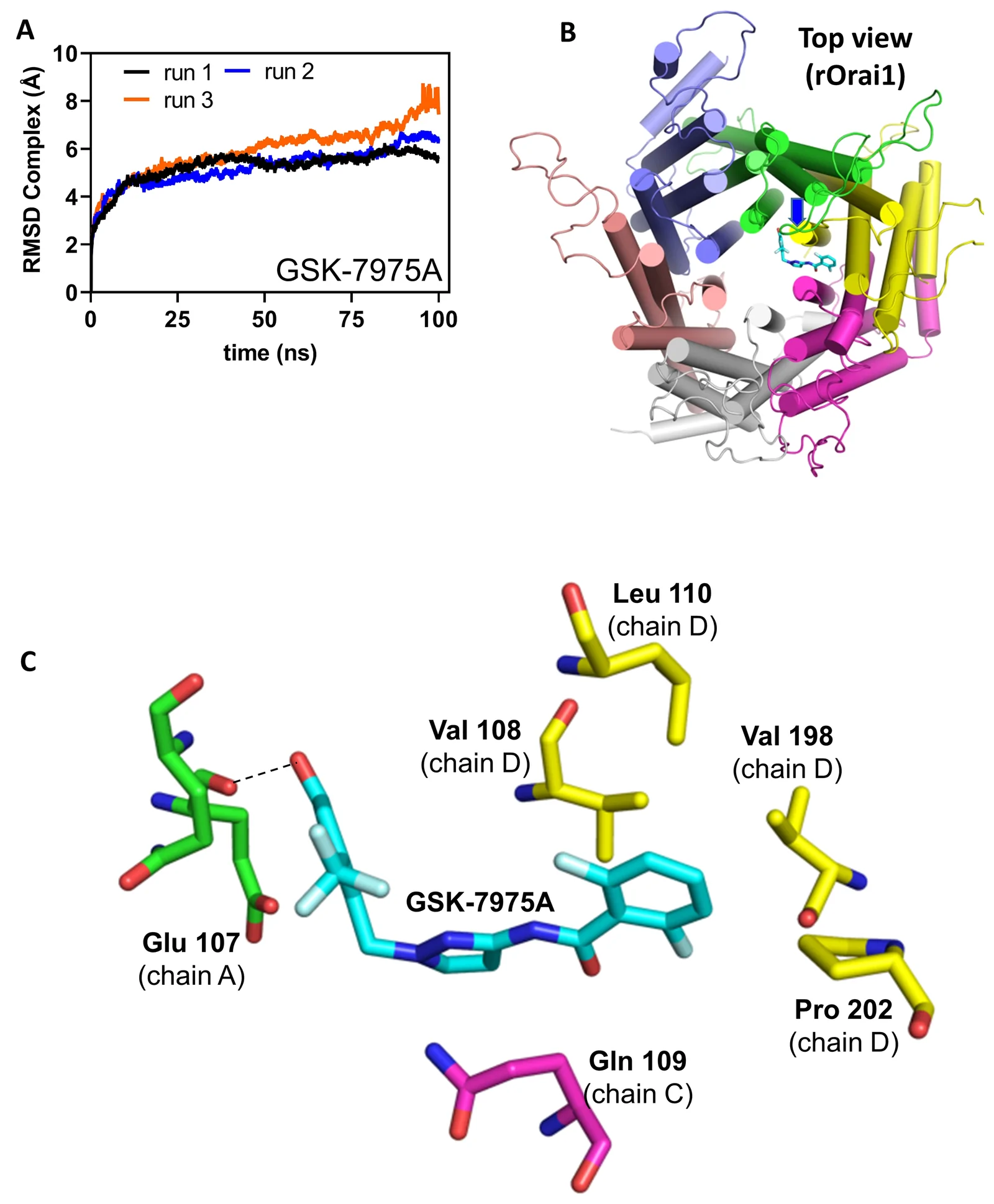
*In silico* prediction of probable binding site and binding mode of GSK-7975A on the extracellular face of rOrai1 hexamer. (A) RMSD plot of GSK-7975A-rOrai1 docked complex obtained from trajectory analyses of 100 ns of MD simulations performed in three independent runs. (B) Top view from the extracellular side showing the most stable pose of GSK-7975A (shown as stick representation) on the extracellular face of the rOrai1 model (shown as cylinders). The pose was obtained from MD simulations using predicted binding mode of GSK-7975A through initial blind docking with AutoDock Vina followed by focused docking using GOLD suite. The ligand pose is shown with a blue arrow. (C) Three-dimensional-ligand interaction profile of the typical best pose of GSK-7975A generated through PLIP server. The ligand and the proximal residues are shown as sticks. Hydrogen bonding interactions are shown with dashed lines. Figures were prepared using PyMOL.

Orientational shifts among these aromatic moieties were observed over the course of the simulation, optimizing ligand stability through enhanced polar and hydrophobic interactions. Specifically, the trifluoromethylphenol group formed a persistent hydrogen bond with Gln109 of one subunit. GSK-7975A also engaged, via hydrophobic interactions, with several residues spanning multiple rOrai1 subunits, including Glu107, Val108, Gln109, Leu110, Val198 and Pro202 (figure 8C). Together, these interactions account for the observed stability of GSK-7975A within this extracellular pocket of rOrai1.

The estimated binding free energy for GSK-7975A at the extracellular site of the rOrai1 hexamer is given in table 1.

#### 2.2.4. Putative binding mode of Synta66 on the rOrai1 hexamer

Like BTP2, Synta66 retains the core aryl amide moiety, but this is flanked by a fluoropyridine moiety and a 2,3-dimethoxyaryl moiety (figure 4A). Unlike BTP2 and GSK-7975A but similar to Pyr6, Synta66 was equally effective in suppressing *I*_CRAC_ when introduced via the bath (extracellular) or pipette (intracellular) solution. We therefore considered both extracellular and intracellular possible binding sites for Synta66 on the rOrai1 hexamer. Initial binding poses were predicted by docking and subsequently refined and assessed for stability using MD simulations done in triplicate.

The predicted extracellular binding site of Synta66 (designated as Synta66_out_) was similar to that of Pyr6_out_, being located within an interhelical pocket formed by two M1 helices from adjacent rOrai1 subunits and one M3 helix from one of these subunits (figure 9B). Analysis of the MD trajectories showed that Synta66_out_ remained largely stable at this extracellular site (figure 9A). Within this pocket, Synta66 underwent modest positional adjustment during the simulation, moving slightly closer to the M3 helix, probably reflecting optimization of electrostatic and van der Waals interactions. PLIP-based analysis (figure 9C) revealed a hydrogen bond between Leu110 of one rOrai1 subunit and the aryl amide nitrogen atom of Synta66. Among other interactions, Pro202 appeared to engage in an arene-π interaction with the 2,3-dimethoxyaryl moiety, while residues Gln109, Leu110, Tyr116, Val198 and Leu201 from adjacent rOrai1 subunits contributed hydrophobic and van der Waals interactions. Together, these non-covalent interactions stabilized the aryl amide moiety in an orientation approximately parallel to the pore axis. The fluoropyridine moiety was inclined towards the pore axis, suggesting targeted positioning of Synta66 within the predicted extracellular interhelical pocket.

**Figure 9.**
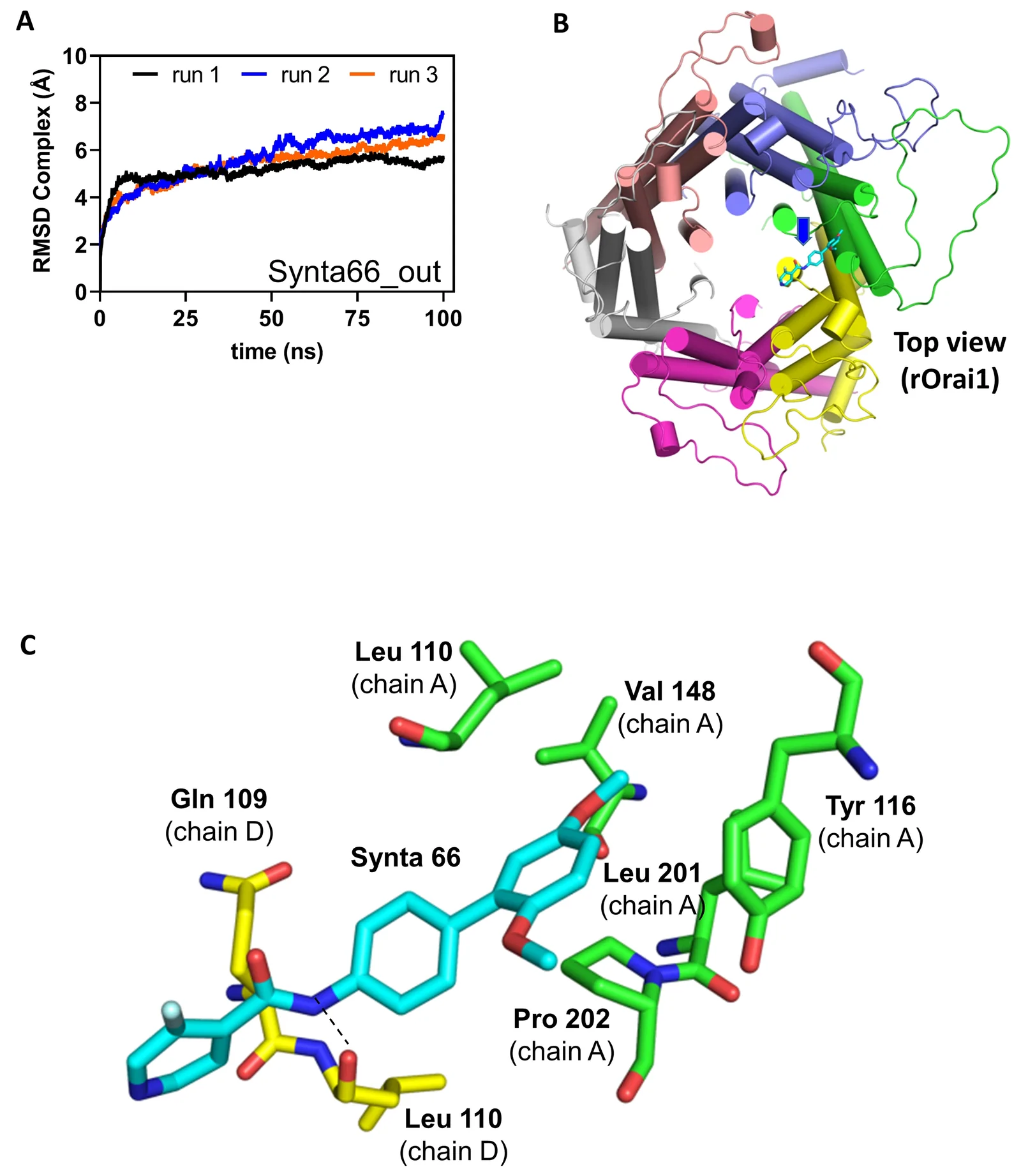
*In silico* prediction of probable binding site and binding mode of Synta66 on the extracellular face of rOrai1 hexamer. (A) RMSD plot of Synta66-rOrai1 docked complex obtained from trajectory analyses of 100 ns of MD simulations performed in three independent runs. (B) Top view from the extracellular side showing the most stable pose of Synta66 (designated as Synta66_out_, shown as stick representation) on the extracellular face of the rOrai1 model (shown as cylinders). The pose was obtained from MD simulations using predicted binding mode of Synta66 through initial blind docking with AutoDock Vina followed by focused docking using GOLD suite. The ligand pose is shown with a blue arrow. (C) Three-dimensional-ligand interaction profile of the typical best pose of Synta66_out_ generated through PLIP server. The ligand and the proximal residues are shown as sticks. Hydrogen bonding interactions are shown with dashed lines. Figures were prepared using PyMOL.

Similar to Pyr6 but unlike BTP2 and GSK-7975A, Synta66 retained its ability to inhibit CRAC channel activity when introduced into the cytoplasm in our whole-cell patch-clamp recordings of RBL-1 cells (figure 4B). This prompted us to predict *in silico* a potential intracellular binding pocket for this molecule on the rOrai1 model.

The second predicted binding pose of Synta66, designated Synta66_in_, occupied a relatively spacious but partially constricted cavity near the cytosolic ends of the M3 and M4 helices (figure 10). During MD simulation, this pocket displayed dynamic flexibility, consistent with local breathing motions. Nevertheless, ligand mobility remained limited because of stabilizing interactions with residues from adjacent rOrai1 subunits, including Thr143, Cys144, Glu174, Leu279, Phe256 and Phe260. The fluoropyridine moiety of Synta66 was buried deep within the pocket, while Thr143 and Cys144 appeared to influence ligand orientation through combined polar, hydrophobic and van der Waals interactions. In addition, the aromatic residues Phe256, His259 and Phe260 further restricted ligand conformational flexibility, thereby stabilizing Synta66 within this putative cytosolic binding pocket (figure 10C).

**Figure 10.**
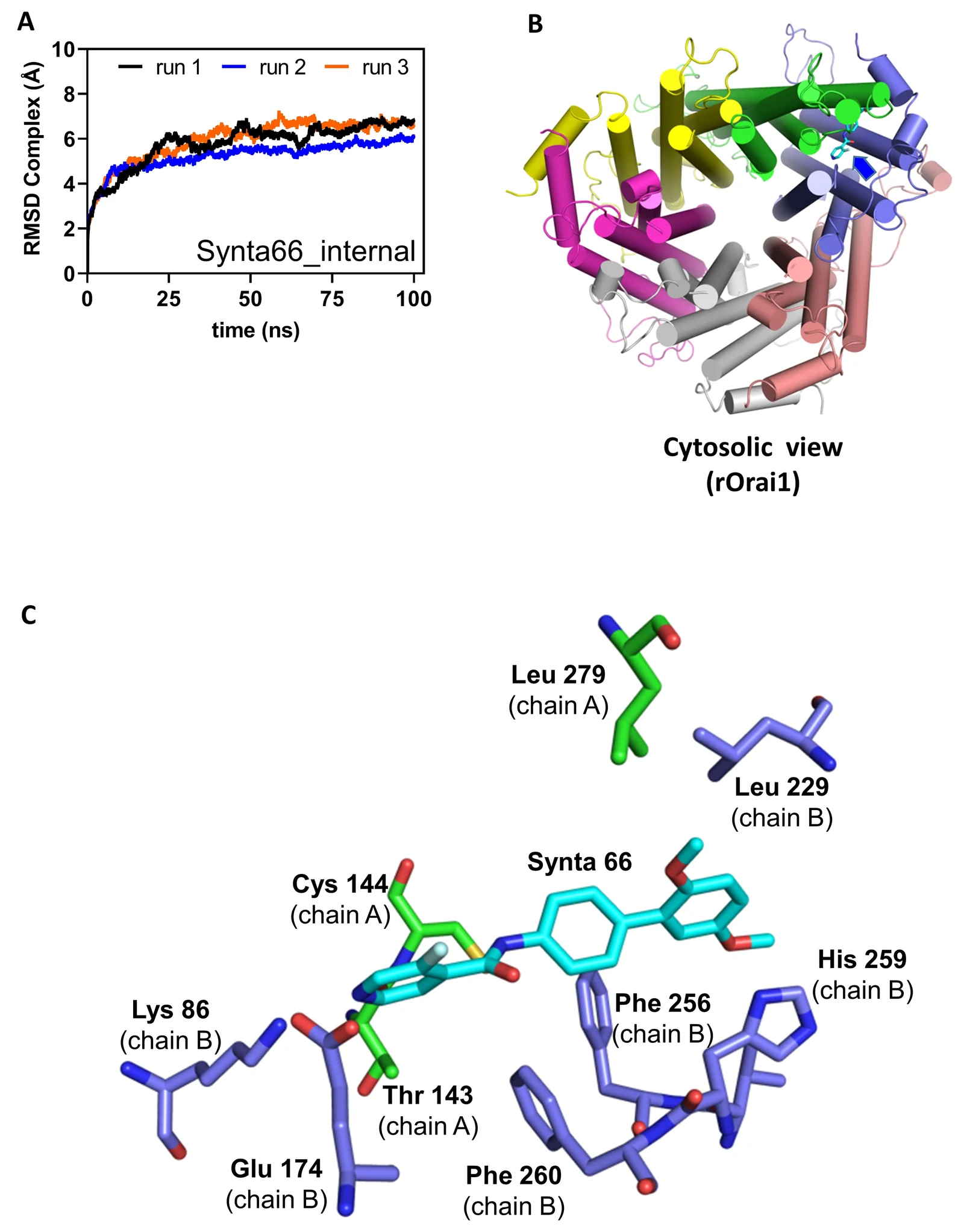
*In silico* prediction of probable binding site and binding mode of Synta66 on the intracellular face of rOrai1 hexamer. (A) RMSD plot of Synta66-rOrai1 docked complex obtained from trajectory analyses of 100 ns of MD simulations performed in three independent runs. (B) Top view from the extracellular side showing the most stable pose of Synta66 (designated as Synta66_in_, shown as stick representation) on the extracellular face of the rOrai1 model (shown as cylinders). The pose was obtained from MD simulations using predicted binding mode of Pyr6 through initial blind docking with AutoDock Vina followed by focused docking using GOLD suite. The ligand pose is shown with a blue arrow. (C) Three-dimensional-ligand interaction profile of the typical best pose of Synta66_in_ generated through PLIP server. The ligand and the proximal residues are shown as sticks. Hydrogen bonding interactions are shown with dashed lines. Figures were prepared using PyMOL.

The estimated energetic profile for Synta66 at both extracellular and intracellular binding pockets is given in table 1.

Across the selected CRAC channel inhibitors, a small number of common residues appear to be involved in their recognition at the extracellular face of rOrai1. These include Val108, Gln109, Leu110 and Val198 (corresponding to Val107, Gln108, Leu109 and Val197 in hOrai1, respectively). These residues reside just above the pore-forming M1 helix and project extracellularly [31]. Notably, these residues lie in close proximity to the highly conserved glutamate residue, Glu107 in rOrai1, which is critical for Ca^2+^ selectivity, pore diameter regulation, pH sensitivity and Ca^2+^-dependent inhibition [1,2]. Intriguingly, a previous study by Romanin and colleagues showed that mutating this glutamate to aspartate, besides reducing Ca^2+^ selectivity, also abolished hOrai1 inhibition by GSK-7975A [13]. In this context, our finding that GSK-7975A inhibits *I*_CRAC_ only when applied extracellularly is entirely plausible, and our proposed ‘site I’ binding mode for GSK-7975A strongly complies with their finding. However, this also suggests that our predicted ‘site II’ binding mode is likely to be unrealistic for this molecule.

Another recent study using photo-crosslinkable derivatives of GSK-7975A provided evidence for direct association of these probes with hOrai1, although the crosslinked residue(s) could not be identified by mass spectrometry [15]. Through docking, the authors in that study suggested two possible ligand-binding regions, including a site associated with the extracellular loops between Orai1 subunits and a second site deeper within the channel pore; however, mutagenesis of residues W76 and Y80 did not support the latter pose [15]. Thus, their results are compatible with, but do not experimentally define, the extracellular binding region proposed here. Our predicted M1/M3 interhelical pocket places GSK-7975A close to the extracellular selectivity-filter region and therefore provides a more specific structural hypothesis that is also consistent with the sensitivity of GSK-7975A inhibition to Orai1 pore geometry reported previously [13].

Like GSK-7975A, BTP2 was also effective only when applied extracellularly in our experiments. This observation is consistent with earlier work in Jurkat T cells, where an extracellular mode of action for BTP2 was proposed [21]. In our predicted binding mode, BTP2 did not interact directly with the conserved Glu107 of rOrai1 but instead engaged the adjacent Gln109. Thus, although BTP2 may not directly contact the selectivity-filter glutamate, its predicted binding site remains in close proximity to this functionally critical region. Val198 was another common residue involved in the extracellular binding modes of all four inhibitors. Given its location on the M3 helix, immediately behind the pore-forming M1 helix, interaction with Val198 may help stabilize ligands at the extracellular interhelical pocket. This predicted binding site is therefore compatible with the extracellular mode of action of BTP2 observed both in our study and in previous reports [21].

Despite their structural similarity to BTP2 and GSK-7975A, the other two CRAC inhibitors, Pyr6 and Synta66, were effective when applied either extracellularly or intracellularly.

Their predicted extracellular binding modes resembled those of BTP2 and GSK-7975A, involving interactions with residues around the extracellular M1/M3 interhelical pocket, including Gln109 of rOrai1. This residue lies adjacent to the conserved Glu107 of rOrai1, corresponding to Glu106 in hOrai1, which is essential for channel selectivity. In addition to this extracellular site, we further propose that Pyr6 and Synta66 may act via distinct intracellular sites, consistent with suggestions made previously that some CRAC inhibitors may act via intracellular sites [6].

Our predicted extracellular binding mode for Synta66 is broadly consistent with that proposed by Waldherr *et al.* [14], who implicated the extracellular TM1/TM3 and loop 1/loop 3 region close to the Orai1 selectivity filter, supported by mutagenesis and functional analysis. In our model, Synta66 similarly occupies an extracellular M1/M3 interhelical pocket close to the selectivity-filter region. Thus, the two studies support a common extracellular region for Synta66 action, although the precise ligand pose and residue-level interactions differ.

It is also worth noting that, for each CRAC inhibitor, we considered only a 1 : 1 stoichiometry during blind docking, in keeping with previous studies [12] reporting Hill coefficients of approximately 1 for some of these inhibitors (GSK-7975A and Synta66). Nonetheless, we cannot rule out multiple occupancies of these inhibitors, for which an experimentally solved inhibitor-bound Orai structure will ultimately be necessary.

## 3. Materials and methods

### 3.1. Cell culture

Rat basophilic leukemia-1 (RBL-1, ATCC: CRL-1378™) cells were grown in Dulbecco's modified Eagle's medium (DMEM) supplemented with 10% (v/v) fetal calf serum, 2 mM L-glutamine and 1% (v/v) penicillin/streptomycin, as previously described [18,32].

### 3.2. Patch clamp electrophysiology

Native CRAC currents (*I*_CRAC_) were recorded from RBL-1 cells at room temperature (25°C) using the patch-clamp technique in its whole-cell configuration. Preparation of the patch pipettes, the composition of the internal (pipette) and extracellular (bath) solutions and recording protocol (including leak subtraction) were as described previously [18]. Briefly, currents were acquired through Axopatch 200B and Digidata 1440 A (molecular devices) in response to voltage ramps (−100 to +100 mV in 150 ms) applied every 2 s; data were digitized at 10 kHz and filtered at 1 kHz and pCLAMP 10 suite (molecular devices) was used for data acquisition and analysis. Peak *I*_CRAC_ amplitude (pA) was normalized to cell size represented by the capacitance (pF) and expressed as current densities (pA pF^−1^). For experimental evaluation of the CRAC channel inhibitors, cells were pretreated with blockers for approximately 20 minutes prior to recording.

### 3.3. Molecular modelling and docking

Homology modelling of rat Orai 1 (rOrai1, Uniprot ID: Q5M848, CRCM1_RAT) was performed through SwissModel server (https://swissmodel.expasy.org/) [33], using published structure of *Drosophila* Orai (DmOrai, PDB ID: 4HKR) as the template. The first 51 residues of rOrai1 sequence seem to be intrinsically disordered as predicted by the AlphaFold [34], (https://alphafold.ebi.ac.uk/entry/Q5M848 and supplementary material, figure S1); therefore, we excluded this portion from the sequence of rOrai1 before modelling. Initially, a dimeric form of rOrai1 model was subjected to refinement through the GalaxyRefine2 server [29], and finally it was assembled into a hexamer based on the published hexameric DmOrai structure (PDB ID: 4HKR). The quality of the final rOrai1 model was assessed through the structure assessment web server [35].

The three-dimensional structures of all the four CRAC channel inhibitors used in this study were obtained from PubChem [36], and their putative binding poses were predicted through *in silico* docking as described previously [25,26]. Briefly, for each molecule, an initial blind docking was performed in three independent runs using AutoDock Vina [27] version 1.2.5 with a GRID box encompassing the entire rOrai1 hexamer. The best ranked and reproducible pose of each molecule from such blind docking was then used as the reference pose for subsequent pose refinement through focused docking (10 independent runs for each molecule) using GOLD [28] version 5.3 suite (CCDC, Cambridge) using the default ChemPLP score. The most reproducible ligand poses among the highest ranked ones were then subjected to further refinement through MD simulation. Integration of such simulation approach with molecular docking allows further refinement of the initial pose as well as evaluation of pose stability and energetics of binding, taking protein structural flexibility in explicit solvent and membrane environment (if relevant for the target) into account. Together, these machine learning approaches often can improve the success rate in virtual screening campaign and results in the identification of novel hits [37,38].

### 3.4. Molecular dynamics simulation and molecular mechanics/Poisson–Boltzmann surface area free energy calculation

#### 3.4.1. System assembly

Membrane-embedded all-atom systems were constructed with CHARMM‑GUI Membrane Builder [39,40]. Protonation states of ionizable residues were assigned automatically for pH 7.4 using PROPKA [41], and the protein termini were replaced independently with an acetyl cap at the N‑terminus and an N‑methylamide cap at the C‑terminus to mitigate artefactual end effects. The ligand structures were parametrized using the CGenFF [42], with topology and parameter files generated. No explicit water molecules were introduced at this stage. Each system was inserted into a fully hydrated POPC bilayer. The systems were neutralized and brought to physiological salinity (150 mM) by random substitution of water molecules with Na^+^ and Cl^−^. All biochemical entities, such as protein, lipids, water and ions, were described by the CHARMM36m force field [43] in conjunction with the TIP3P water model [44].

#### 3.4.2. Molecular dynamics simulations

All simulations were carried out with GROMACS 2023.3 [45] on NVIDIA A4000 GPUs. After a six-stage equilibration comprising 400 ps of restrained NVT followed by 1.5 ns of restrained NPT, each system was advanced for 100 ns in the isothermal–isobaric ensemble. Temperature was held at 303.15 K and pressure at 1 bar. A leap-frog integrator with a 2 fs time-step was used, and all bonds involving hydrogen were constrained by the LINCS algorithm [46] to enable stable integration. Neighbour lists were constructed with the Verlet cut-off scheme, extending to 1.2 nm [47]. Lennard-Jones forces were switched linearly between 1.0 and 1.2 nm, while long-range Coulomb interactions were treated with particle-mesh Ewald [48] employing the same real-space cut-off. Pressure was controlled semi-isotropically with the stochastic cell-rescale barostat, using a 5 ps relaxation time and a compressibility of 4.5 × 10^–5^ bar^–1^. For every system, three statistically independent replicas were initiated from distinct Maxwell–Boltzmann velocity distributions, yielding a total sampling of 300 ns. Coordinates were saved every 50 ps, and energy as well as virial terms were recorded every 2 ps. No centre-of-mass restraints were imposed, and long-range dispersion corrections were deliberately omitted, ensuring unbiased characterization of the membrane-embedded channels. Each production simulation was run for 100 ns, with three independent replicates per system. Across all systems, this yielded an aggregate simulation time of 1.8 µs.

#### 3.4.3. Analysis of simulation trajectories

Structural and energetic analyses were conducted using in-house scripts. Trajectories were parsed with MDAnalysis [49] along with MDTraj [50], and RMSDs were evaluated every 10 ps to ensure robust convergence. Binding free energies were quantified with gmx_MMPBSA v. 1.6.2 [51,52], which processed 100 snapshots extracted from each replica. Frames 8000 to 10 000 were taken at an interval of 20, corresponding to the 80–100 ns portion of production phase. The calculation employed the CHARMM‑specific charmm_radii parameter set, thereby assigning Poisson–Boltzmann atomic radii consistent with the CHARMM36m force field [43]. Electrostatic solvation was modelled with a solute dielectric constant of 2, a solvent dielectric constant of 80, an ionic strength of 150 mM, and a fill ratio of 4.0; atomic radii from the topology were used for both the Poisson–Boltzmann and non-polar terms. Per residue energy decomposition was performed, generating detailed contributions for every amino acid situated within 4.5 Å of the ligand. All the systems went through the same analysis protocol.

### 3.5. Analysis of ligand–protein interaction

For each stable docked complex refined through MD simulation, the three-dimensional ligand interaction profiles were deduced through the PLIP [53]. All relevant figures were generated in PyMOL Molecular Graphics System (version 1.8.2.0 Open-Source, Schrödinger, LLC) while ligand interaction figures were obtained from PLIP web tool [53].

Amino-acid numbering throughout the molecular modelling and docking analyses refers to rOrai1 (UniProt Q5M848). Where relevant, the corresponding hOrai1 residues are provided on first mention only.

### 3.6. Statistical analysis

Results were expressed as means ± s.e.m. for *n* independent experiments done at least in triplicates. Statistical comparisons of the mean values were performed using GraphPad Prism 8 (GraphPad software Inc., CA, USA). One way ANOVA followed by Dunnett's test was used to compare the groups with respect to the control group. *P*-values less than 0.05 was considered to be significant.

## 4. Conclusion

This study combined whole-cell patch-clamp recordings of *I*_CRAC_ in RBL-1 cells with *in silico* docking and MD simulations to reassess the mode of action of four widely used CRAC channel inhibitors. Although these compounds share broadly comparable physico-chemical properties, they displayed distinct sidedness of action: BTP2 and GSK-7975A inhibited *I*_CRAC_ only from the extracellular side, whereas Pyr6 and Synta66 were effective from both sides of the PM.

Our modelling suggests that these inhibitors are likely to bind at pockets located near the pore-forming M1 helices of the rOrai1 hexamer. Their putative extracellular binding sites involve residues close to the conserved selectivity-filter glutamate, Glu107 in rOrai1 and Glu106 in hOrai1. This suggests that these compounds may inhibit CRAC channel activity by altering pore geometry, restricting M1 helix rearrangements and/or impairing pore dilation. These findings and hypotheses align well with suggestions from previous studies [13,14]. For Pyr6 and Synta66, the predicted intracellular poses provide a plausible explanation for their cytosolic activity. Together, these findings support the idea that structurally related CRAC channel inhibitors can act through distinct, and potentially multiple, binding modes.

Because this study used endogenous CRAC channels in RBL-1 cells, future work should validate the predicted binding residues by site-directed mutagenesis in defined Orai isoform backgrounds.

An important limitation of the present study is that electrophysiological experiments were performed using endogenous CRAC channels in RBL-1 cells, whereas our computational analyses were based on a rat Orai1 hexamer. Since endogenous CRAC currents may involve contributions from multiple Orai isoforms, and Orai1, Orai2 and Orai3 can exhibit distinct pharmacological profiles [54,55], the inhibitor sensitivity observed here cannot be unequivocally attributed to Orai1. Future studies using defined Orai isoforms, ideally expressed individually in an Orai-null background together with site-directed mutagenesis of the predicted binding residues, will therefore be important for experimentally validating the proposed Orai1 binding modes.

## Supplementary Material



## Data Availability

The data supporting the findings of this study are publicly available in the Zenodo repository [56]. Supplementary material is available online [57].
