# Supplementary material for: Structurally related Ca2+-release activated Ca2+ channel inhibitors exhibit distinct extracellular and intracellular modes of action
Source: R Soc Open Sci. 2026 Nov 11;13(11):261544. doi: 10.1098/rsos.261544 (PMC13750932; doi:10.1098/rsos.261544)
Supplement: Supplementary file 1 [file rsos.261544.zip › rsos.261544/rsos261544_si_001.docx]

# Supporting information

**Supplementary Figure 1. Cartoon representation of the rat Orai 1 single subunit model from AlphaFold.** The model was retrieved from AlphaFold (<https://alphafold.ebi.ac.uk/entry/Q5M848>) and the disordered region is highlighted in red colour. The figure was generated in PyMOL Molecular Graphics System (version 1.8.2.0 Open-Source, Schrödinger, LLC).

**Supplementary Figure 2. Ramachandran Plot of rat Orai1 hexameric model used for docking.** The plot was generated through structure assessment webserver (<https://swissmodel.expasy.org/assess>).

**Supplementary Table 1. Predicted physicochemical properties of the CRAC channel inhibitors used in the study**

|  | **BTP2** | **Pyr6** | **Synta66** | **GSK-7975A** |
| --- | --- | --- | --- | --- |
| MW (g/mol) | 421.3 | 418.27 | 352.4 | 397.3 |
| XlogP3 | 3.8 | 3.6 | 3.4 | 3.7 |
| TPSA ((Å)^2^) | 101 | 59.8 | 60.4 | 67.2 |
| No. of heavey atoms | 28 | 29 | 26 | 28 |
| No. of H-bond donors | 1 | 1 | 1 | 2 |
| No. of H-bond acceptor | 11 | 10 | 5 | 8 |
| No. of Rotatable bond | 3 | 3 | 5 | 4 |

*MW, Molecular Weight; XlogP3, predicted octanol-water partition coefficient; TPSA, total polar surface area. Values are predicted and taken from PubChem ( <https://pubchem.ncbi.nlm.nih.gov/>).

*MW, Molecular Weight; XlogP3, predicted octanol-water partition coefficient; TPSA, total polar surface area. Values are predicted and taken from PubChem ( <https://pubchem.ncbi.nlm.nih.gov/>).
